# Rectal seed bezoar due to sunflower seed: a case report and review of the literature

**DOI:** 10.11604/pamj.2018.31.157.12539

**Published:** 2018-10-31

**Authors:** Dimitrios Manatakis, Maria Sioula, Ioannis Passas, Helen Zerbinis, Christos Dervenis

**Affiliations:** 1 Department of Surgery, Konstantopouleio General Hospital "Agia Olga", Nea Ionia, Athens, Greece

**Keywords:** Bezoar, phytobezoar, seed

## Abstract

Seed bezoars are a subcategory of phytobezoars, caused by consumption of indigestible vegetable or fruit seeds. We present the case of a 64-year-old male patient, who presented at the Emergency Department, complaining of constipation, tenesmus and rectal pain. History and digital examination revealed a rectal seed bezoar due to sunflower seeds, impacted in the lower rectum. The patient underwent manual disimpaction under general anaesthesia, after conservative measures failed. Seed bezoars represent a different pathophysiological process compared to fibre bezoars. They are usually found in the rectum of patients without predisposing factors, causing constipation and anorectal pain. History taking and digital rectal examination are the cornerstones of diagnosis, with manual disimpaction under general anaesthesia being the procedure of choice.

## Introduction

Bezoars are retained concretions of indigestible foreign material that accumulate and conglomerate in the gastrointestinal tract lumen [1, 2]. Most bezoars are caused by ingestion of hair (trichobezoar), fruit and vegetable fibres (phytobezoar), undigested milk concretions (lactobezoar) or medications (pharmacobezoar) [1, 2]. Seed bezoars are a subcategory of phytobezoars, caused by undigested vegetable seeds or fruit pits. In contrast to other bezoar categories, the majority is found in the rectum of patients with no predisposing factors [3, 4]. We hereby present our experience with a case of a rectal bezoar due to sunflower seeds, causing faecal impaction in an otherwise healthy adult patient, and a brief review of the literature on gastrointestinal seed bezoars.

## Patient and observation

**Figure 1:**
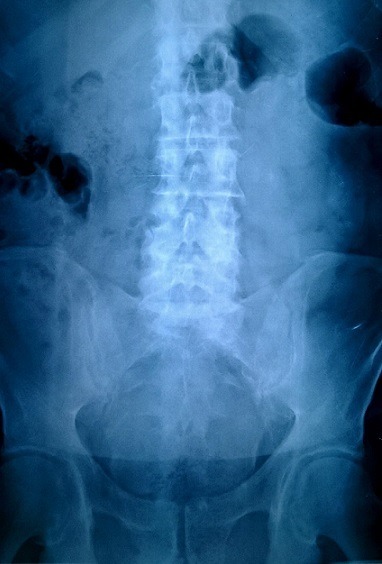
Abdominal X-ray showing a faecal mass in the rectum

**Figure 2:**
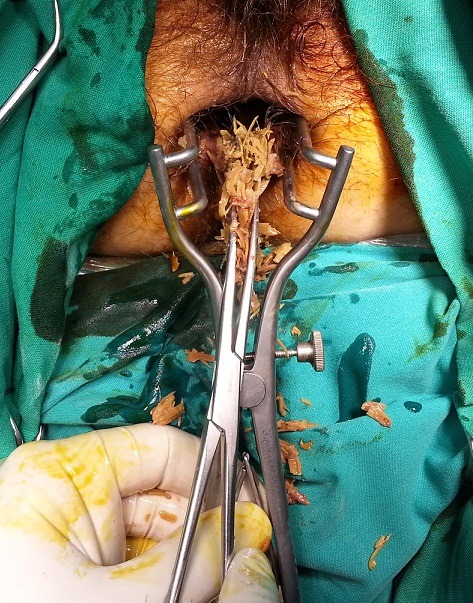
Manual disimpaction of the sunflower seed bezoar in the operating theatre under general anaesthesia

A 64-year-old man presented at the emergency department with a 24-hour history of faecal impaction, tenesmus and rectal pain. The patient reported consumption of large quantities of unshelled sunflower seeds over the past week. On admission, physical examination revealed a soft, non-tender abdomen, with no peritoneal signs. Digital rectal examination revealed a large phytobezoar with sharp edges, impacted in the distal rectum. Basic laboratory tests were all within normal range and an abdominal X-ray showed a faecal mass in the pelvis (Figure 1). Flexible rectoscopy confirmed the impacted sunflower seed bezoar and additionally revealed microlacerations of the rectal mucosa due to the sharp debris, but the endoscope failed to pass beyond the bezoar mass. Multiple attempts at removal with conservative mo-dalities (including manual disimpaction, enemas, rectal tube) resulted in little success. The patient was taken to the operating theatre, where he underwent manual extraction of the bezoar under general anaesthesia (Figure 2). Enemas and stool softeners were administered twice daily over the following two days, to facilitate defecation of residual husks. His postoperative course was uneventful and he was discharged from hospital on the 3^rd^ postoperative day. At 30-day follow-up, the patient reported normal bowel movements and complete remission of anorectal discomfort.

## Discussion

Bezoars can occur anywhere in the gastrointestinal tract, from the oesophagus to the rectum, however they are most commonly found in the stomach [1, 2]. Recognised risk factors are prior gastric surgery, neuropsychiatric disease, endocrinopathies impairing gastrointestinal motility and poor mastication [1, 2]. Seed bezoars are caused by the accumulation of indigestible vegetable or fruit seeds in the intestinal lumen. Grains and seeds pass through the pylorus and the ileocaecal valve, due to their small size, and accumulate gradually in the colon. Reaching the rectum, the faecal mass is further dehydrated and forms a hard bezoar, usually presenting as faecal impaction. On the contrary, the typical gastric phytobezoar is caused by consumption of indigestible fibres (cellulose, lignin, tannins). These fibres, contained in the skin of fruits and vegetables, polymerise and agglutinate in the acidic environment of the stomach, and form a glue-like coagulum which affixes to other material and only rarely passes through the pyloric sphincter [5]. Rectal seed bezoars seem to arise in patients with no risk factors [3, 4]. Eitan *et al* identified risk factors only in 7.2% of patients with seed bezoars, compared to 80.9% of patients with seedless faecal impaction [3, 4]. Large retrospective series of gastric and intestinal bezoar cases, on the other hand, reported rates of risk factors exceeding 85% (43-55% incidence of prior gastric surgery, 12-28.6% diabetes, persimmon consumption 17.5-40.5%) [2, 5-7]. Whereas gastric bezoars may run asymptomatic for years or present with vague, non-specific symptoms, most seed bezoars occur in the rectum and cause constipation or non-specific abdominal or rectal pain [8-10]. Intestinal obstruction is relatively infrequent and mainly affects patients with seed bezoars in the terminal ileum [11, 12]. While bowel perforation is the most feared complication, peritonitis was reported only in one case [13]. Diagnosis of rectal seed bezoars should be suggested by a careful history and con-firmed by digital rectal examination. Plain abdominal radiographs may show a solid stool mass, but generally they are within normal limits, as in our case. Computerised tomography scans may be helpful, excluding intestinal obstruction and revealing the impacted bezoar [14]. In adult patients, a full colonoscopy is advisable, once the rectum is evacuated, to exclude malignant strictures of the colon. In children with ambiguous symptomatology, negative imaging studies and suspicion of acute appendicitis, a digital rectal examination should not be omitted. Revealing a rectal seed bezoar practically eliminates the need for laparotomy [4, 15]. Rectal seed bezoars are best treated by manual evacuation under general anaesthesia, to minimise patient discomfort, while surgery is practically inevitable for small bowel seed bezoars presenting as intestinal obstruction [6]. Depending on the intraoperative findings, the surgeon may choose between enterotomy and removal of the obstructing bezoar, fragmenta-tion and milking of the bezoar through the ileocaecal valve, and segmental enterectomy. Conservative treatment appears to be more efficient in patients with gastric fibre bezoars, in whom chemical dissolution combined with endoscopy has been very successful [6, 16, 17]. On the other hand, very few cases of rectal seed bezoars are amenable to conservative measures (fleet enemas, stool softeners) [3, 4]. Moreover, endoscopy alone usually fails to extract the bezoar, since the endoscope cannot pass beyond the seed mass without risking perforation of the rectum [3, 4].

## Conclusion

Seed bezoars are a subgroup of phytobezoars, usually found in the rectum of patients without predisposing factors, causing constipation and non-specific abdominal or anorectal pain. Careful history and digital rectal examination are the sine-qua-non of diagnosis, in combination with rectoscopy and CT scans in selected cases. The majority of rectal seed bezoars can be manually disimpacted under general anaesthesia to minimise pain and discomfort. Intestinal seed bezoars on the other hand are usually found in the terminal ileum causing intermittent obstruction and require therefore surgical intervention.

## Competing interests

The authors declare no competing interests.
